# Chemical Characterization and Hypoglycaemic Activities In Vitro of Two Polysaccharides from *Inonotus obliquus* by Submerged Culture

**DOI:** 10.3390/molecules23123261

**Published:** 2018-12-10

**Authors:** Jiao Xue, Shisheng Tong, Zhaorun Wang, Ping Liu

**Affiliations:** 1 College of Food Science and Nutritional Engineering, China Agricultural University, Beijing 100083, China; xj1154009143@163.com; 2 Bio-Pharmaceutical College, Beijing City University, Beijing 100094, China; shishengt@163.com; 3 School of Life Sciences, Inner Mongolia University, Inner Mongolia 010021, China; 15949783470@163.com

**Keywords:** *Inonotus obliquus*, submerged culture, polysaccharides, diabetes, HepG2 cells

## Abstract

Polysaccharides from the fungus *Inonotus obliquus* have been found to be biologically active. In this study, we carried out a preliminary characterisation and assessment of the hypoglycaemic activities of the polysaccharides (IOEP) from *Inonotus obliquus* obtained by liquid fermentation. Two polysaccharides, IOEP1 and IOEP2, were isolated from IOEP. IOEP1, with a molecular weight of 20 KDa, was mainly composed of galatose and mannose, while IOEP2, with a molecular weight of 200 KDa, was mainly composed of arabinose. Fourier-transform infrared analysis showed that both IOEP1 and IOEP2 were pyran-type polysaccharides. ^1^H-NMR spectra showed that the glycosidic bonds of IOEP1 and IOEP2 were both α-type and β-type. In addition, IOEP1 and IOEP2 strongly increased the glucose consumption of HepG2 cells and insulin-resistant HepG2 cells in vitro. These findings provide a theoretical basis that IOEP1 and IOEP2 might be suitable as anti-diabetes agents in functional foods and natural drugs.

## 1. Introduction

The epidemic of diabetes mellitus (DM) is rapidly becoming a global health concern, posing a huge economic burden in both developed and under-developed countries [1]. Diabetic patients tend to develop complications such as cardiovascular disease, retinopathy, and nephropathy [2,3,4]. The treatment of hyperglyacemia or hyperlipidaemia in diabetes involves diet control, exercise, and the use of supplements or drugs against hyperglyacemia or hyperlipidaemia [5]. However, oral medicines for such purposes have numerous and serious adverse effects [6,7]. The management of hyperglyacemia or hyperlipidaemia with minimal side effects in clinical experiments and relatively low costs remains a challenge in medicine [5]. Therefore, it is of great importance to study and develop hypoglycaemic drugs with high efficiency and low toxicity, particularly the drugs extracted from natural resources, which have no or minimal side effects [8].

*Inonotus obliquus* (Pers.: Fr.) Pil., a higher basidiomycetes mushroom, is a rare medicinal mushroom belonging to the family of Hymenochaetaceae Donk. It is parasitic in nature and is distributed in the Russian Far East, northeast China, and some other countries at latitudes of 45–50° N [9]. This mushroom has been used in folk medicine for cancer treatment for more than four centuries in Russia and western Siberia [10,11,12]. In recent years, it has been widely studied for its health-promoting properties and relatively low toxicity [13]. This rare medicinal macrofungus has been documented to contain polysaccharides, polyphenols, triterpenes, melanin, and steroids, showing an array of biological activities [14,15,16]. Animal and clinical experiments have shown that *I. obliquus* can be used as a drug to prevent and cure cancer, cardiopathy, diabetes, AIDS (acquired immunodeficiency syndrome), and other diseases [17,18]. Chemical analysis shows that *I. obliquus* contains more than 200 kinds of active substances [19,20,21,22], Diao etal [23] found that *I. obliquus* polysaccharides have therapeutic effects against diabetes via multiple pathways, including antioxidative effects.

Most *I. obliquus* populations are parasitic on birch trees and exist in the form of fruiting bodies. Because the natural fruiting body takes 10–15 years to attain significant medicinal value, artificial techniques such as liquid fermentation are being used to grow the mycelia *I. obliquus*. This method has the advantages of shorter production cycles, higher yields, and no seasonal environment restriction. Submerged fermentation is an effective process for producing biomass and bioactive compounds, especially exo-polysaccharides. Recently, we validated the optimisation of a submerged fermentation for the production of bioactive polysaccharides from *I. obliquus* using the orthogonal test.

The aim of the present work was to obtain extracellular polysaccharides with homogeneous components from *Inonotus obliquus* in submerged fermentation, as well as to reveal its structural characterization and evaluate its hypoglycaemic activity.

## 2. Results

### 2.1. Determination of Molecular Weight (Mw) Distribution

**Figure 1:**
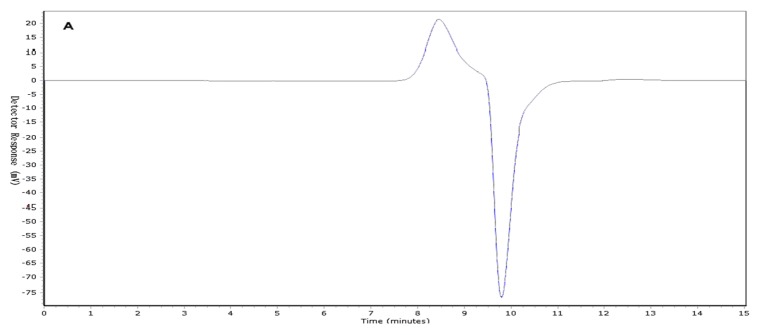

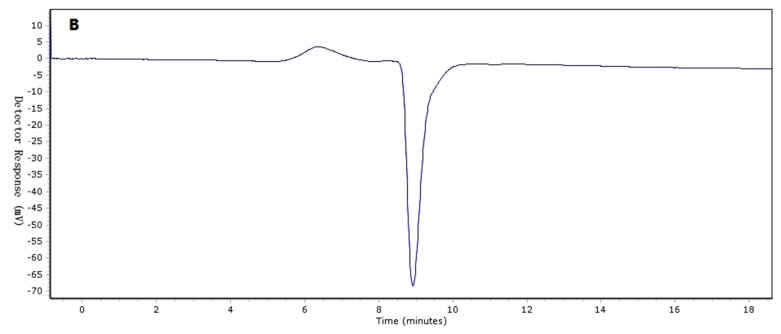
(**A**) The gel permeation chromatography (GPC) of *I. obliquus* crude polysaccharide (IOEP1); (**B**) the GPC of IOEP2.

Two *I. obliquus* crude polysaccharide (IOEP) fractions were collected, namely IOEP1 (IOEP washed with distilled water) and IOEP2 (IOEP washed with 0.5 mol/L NaCl). As shown in Figure 1, both IOEP1 and IOEP2 showed only a single and symmetrical narrow peak with elution time by gel permeation chromatography (GPC), which indicated that the IOEP1 and IOEP2 were polysaccharides with a relatively concentrated molecular weight distribution. Their average relative molecular weights were 20 KDa and 200 KDa, respectively. The inverted peak in the figure is the solvent peak.

### 2.2. Analysis of Physicochemical Properties and Monosaccharide Compositions

Table 1 shows the contents of the major chemical components of the two polysaccharides. The carbohydrate contents in IOEP1 and IOEP2 were 87.04% and 78.73%, respectively. There were trace amounts of protein.

**Figure 2:**
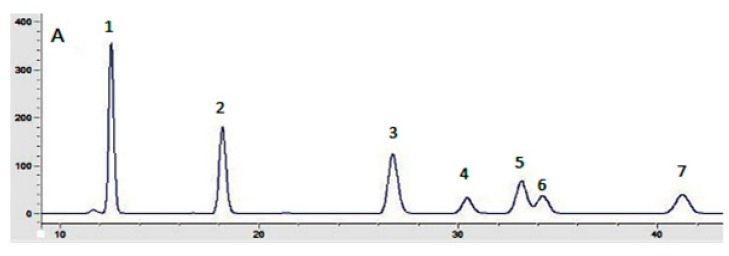

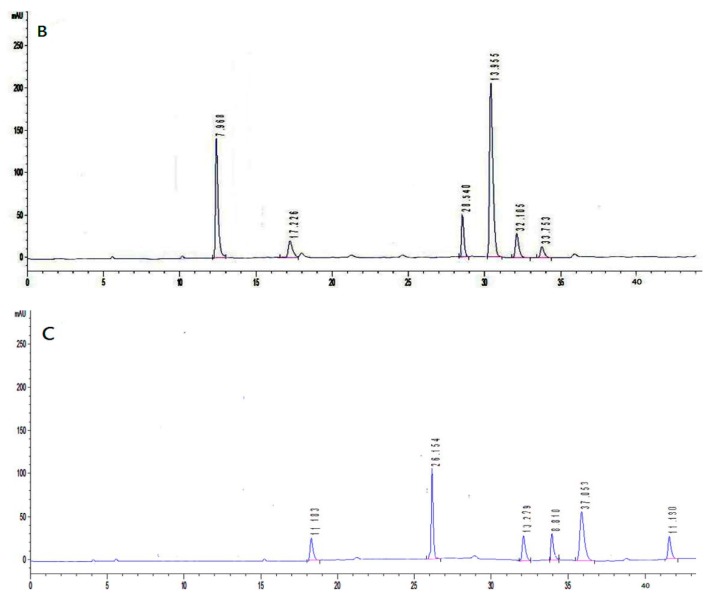
Chromatograms of monosaccharides compositions: (**A**) seven monosaccharides (1: mannose, 2: rhamnose, 3: glucose, 4: galactose, 5: xylose, 6: arabinose, 7: fucose); (**B**) IOEP1; (**C**) IOEP2.

Figure 2 depicts the composition of the monosaccharides (mannose, rhamnose, glucose, galatose, xylose, arabinose, and fucose) of the two polysaccharides. As can be seen, IOEP1 is mainly composed of galatose, mannose, arabinose and glucose, and has no fucose. IOEP2 is mainly composed of arabinose and glucose, but has no mannose.

**Figure 3:**
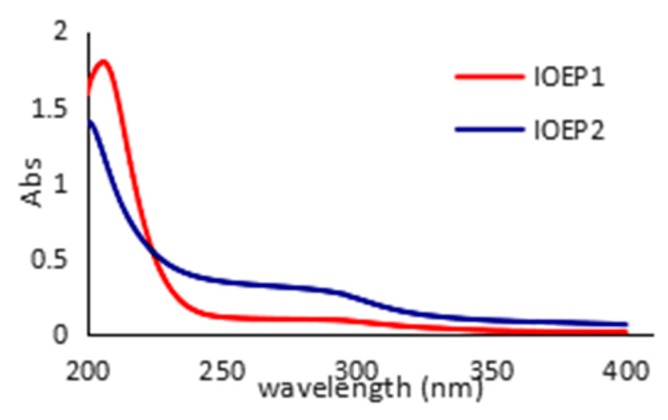
UV absorption spectra of IOEP1 and IOEP2.

In the UV-visible (UV-vis) spectra, the absorption peak at 260 or 280 nm indicated that the samples might contain nucleic acides, proteins, or peptides [24,25]. As depicted in Figure 3, the two polysaccharides showed characteristic peaks of polysaccharides and proteins on the basis of ultraviolet absorption maxima. Different samples had different absorption peaks at different values at 190 nm, and a lower protein absorption peak appeared at around 280 nm. After the removal of protein by Servage treatment, this absorption peak remained, suggesting IOEP1 and IOEP2 contain a certain amount of bound protein.

### 2.3. Fourier-Transform Infrared Spectroscopic Analysis

**Figure 4:**
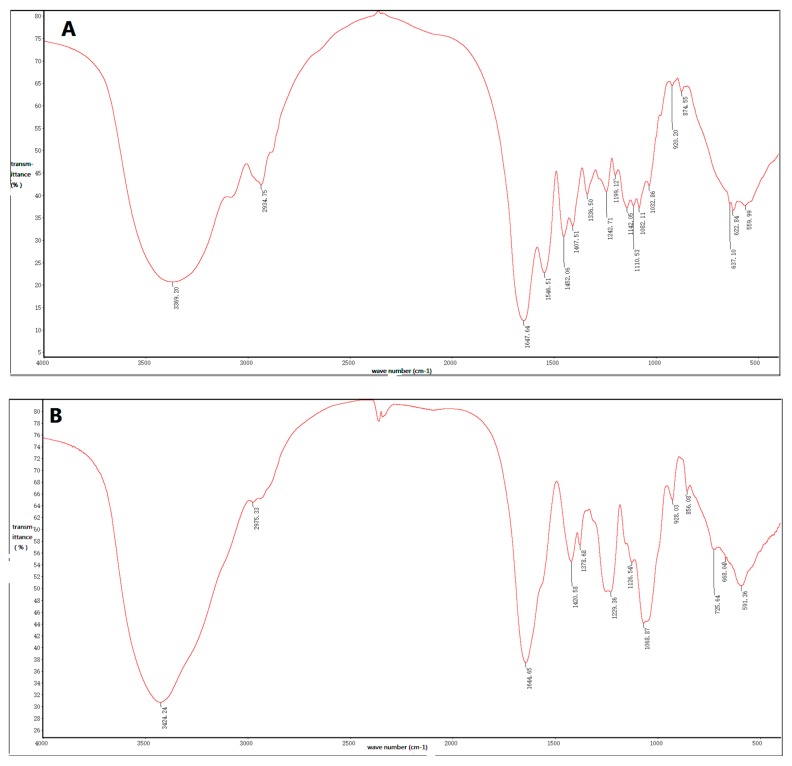
Fourier-transform infrared spectroscopy of (**A**) IOEP1 and (**B**) IOEP2.

IOEP1 and IOEP2 have similar Fourier-transform infrared (FT-IR) absorption bands, indicating similarities in structural features. Figure 4 depicts the typical FT-IR spectrum of IOEP1 and IOEP2 in the 4000 cm^−1^ to 400 cm^−1^ region. According to previous literature, the broad, strongly represented, intense band at 3400 cm^−1^ is due to the stretching vibration of O–H bonds [26,27]. The signal at 3000 cm^−1^ to 2800 cm^−1^ can be associated with the stretching vibration of the C–H bond in the sugar ring [28]. The hydrated hydroxyl bending vibration absorption peak occurred at 1665 cm^−1^ to 1635 cm^−1^. The fingerprint regions that can reflect the monosaccharide types, substituents, and epimers of different sugars are in the range of 1800–700 cm^−1^. Among them, the deformation peak of C–H is in the range of 1500–1200 cm^−1^, and that of C–O–C is in the range of 1150–1000 cm^−1^.

The characteristic absorption peaks of the pyran ring of sugars are in the 1100–1010 cm^−1^ region, and the IOEP1 and IOEP2 have characteristic absorption peaks at 1032.86 cm^−1^ and 1068.87 cm^−1^, respectively. Therefore, it was judged that both polysaccharides were a pyranose form of sugars. The absorption peak near 870 cm^−1^ is the characteristic absorption peak of mannose. From the IR spectra of the two polysaccharides, it was found that IOEP1 has mannose, while IOEP2 does not, which is consistent with the monosaccharide composition data measured by the HPLC (High Performance Liquid Chromatography) method.

### 2.4. ^1^H-NMR Analysis

The structural features of the two polysaccharides were further analysed through the ^1^H-NMR spectrum, which mainly solves the problem of the configuration of glycosidic bonds in the structure of polysaccharides. The chemical shifts at 4.9–5.6 ppm and 4.3–4.9 ppm in the ^1^H-NMR spectrum could be assigned to the typical signals of the anomeric protons of α- and β-anomers, respectively [29]. Moreover, the anomeric protons at 4.9–5.5 ppm indicated that IOEP1 and IOEP2 were mainly composed of several types of sugars [30]. 

**Figure 5:**
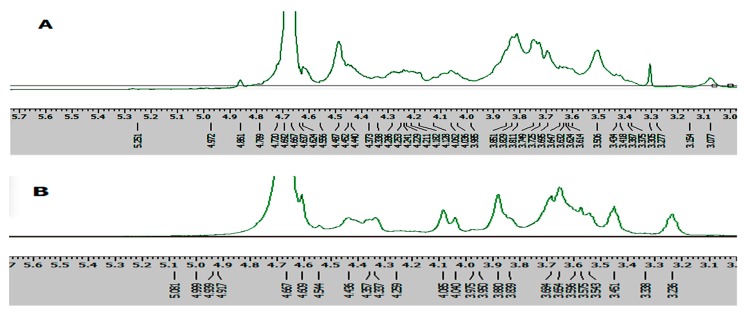
^1^H-NMR spectra of (**A**) IOEP1 and (**B**) IOEP2.

The ^1^H-NMR spectra of IOEP1 and IOEP2 are shown in Figure 5. The peak of the IOEP1 chemical shift at 4.789–4.440 is the peak of proton vibration absorption on the sugar ring, and there is an absorption peak above 4.8 ppm, indicating that the IOEP1 is likely to contain an α-glycosidic bond and a β-glycosidic bond. Similarly, the IOEP2 is also likely to contain an α-glycosidic bond and a β-glycosidic bond.

### 2.5. Hypoglycaemic Activities In Vitro

#### 2.5.1. Cytotoxicity of IOEP1 and IOEP2 at Different Concentrations against HepG2 Cells

Table 2 shows the effect of different concentrations of IOEP1 and IOEP2 on cell viability. The table shows that as the concentration of the polysaccharide increased, the cell survival rate gradually decreased. Compared with the positive control group after 24 h culture, IOEP1 and IOEP2 had little effect on the survival rate of cells at concentrations of 10–80 µg/mL, while some damage to the cells at a concentration of 160 µg/mL occurred, indicating that a high concentration of polysaccharide solution was detrimental to the cells. Thus, we chose polysaccharides at a concentration range of 10–80 µg/mL for further studies on hypoglycaemic activities.

#### 2.5.2. Glucose Consumption Experiment of IOEP1 and IOEP2 on HepG2 Cells

**Figure 6:**
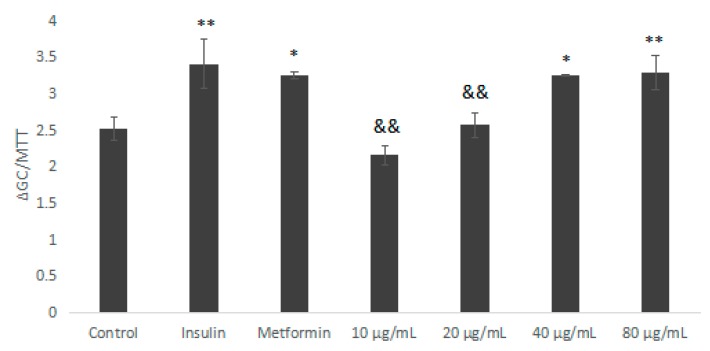
The impact of IOEP1 on the glucose uptake of HepG2 cells. Compared with the control group, * *p* < 0.05, ** *p* < 0.01; compared with the insulin group, ^&&^ *p* < 0.01. GC—glucose concentration; MTT—3-(4,5-dimethyl-2-thiazolyl)-2,5-diphenyltertazolium bromide

Metformin (Met) and insulin are commonly-used hypoglycaemic drugs. As shown in Figure 6, we used Met and insulin as the positive control. Compared with the blank control group, IOEP1 significantly increased the glucose uptake rate of HepG2 cells. The difference was statistically significant (*p* < 0.01, *p* < 0.05). Furthermore, in a certain concentration range (10–80 µg/mL), as the concentration of IOEP1 increased, the tendency to promote the glucose uptake rate of HepG2 cells of IOEP1 first increased and then stabilized. The glucose consumption of cells treated with IOEP1 was significantly higher than that of the control group at 40 µg/mL (*p* < 0.01).

**Figure 7:**
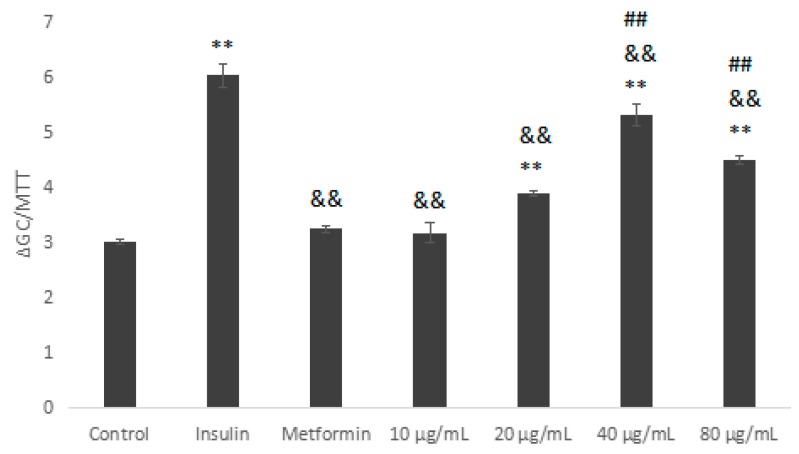
The impact of IOEP2 on the glucose uptake of HepG2 cells. Compared with the control group, ** *p* < 0.01; compared with the insulin group, ^&&^ *p* < 0.01; compared with the Metformin (Met) group, ^##^ *p* < 0.01.

Similarly, the glucose consumption of the IOEP2 sample group (10–80 µg/mL) increased compared with the control group (Figure 7). When the IOEP2 concentration was 40 µg/mL, the glucose consumption was significantly higher than that of the Met group (positive control). In addition, when the concentration of IOEP2 reached 80 µg/mL, the trend of promoting glucose consumption decreased, which may be related to the damage to cell survival at higher concentrations; however, this warrants further study. Nevertheless, IOEP1 and IOEP2 could increase glucose consumption in HepG2 cells at an appropriate concentration range.

#### 2.5.3. Glucose Consumption Assay on Insulin Resistant HepG2 Cells

**Figure 8:**
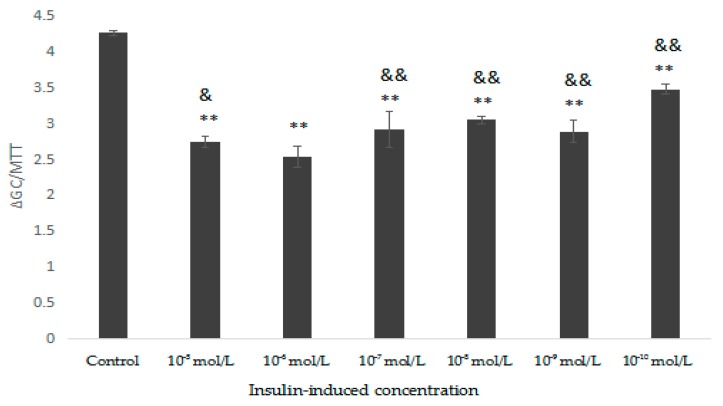
Effect of different concentrations of insulin on HepG2 cells insulin resistance. Compared with control, ** *p* < 0.01; compared with 10^−6^ mol/L, ^&^ *p* < 0.05, ^&&^ *p* < 0.01.

As shown in Figure 8, HepG2 cells were induced with different concentrations of insulin, except the control group, and the glucose consumption was significantly lower than that of control group, indicating that the induction was successful. Compared with the control, when the insulin concentration was 10^−6^ mol/L, the results were the most significant (*p* < 0.01). Therefore, HepG2 cells were induced with 10^−6^ mol/L insulin.

**Figure 9:**
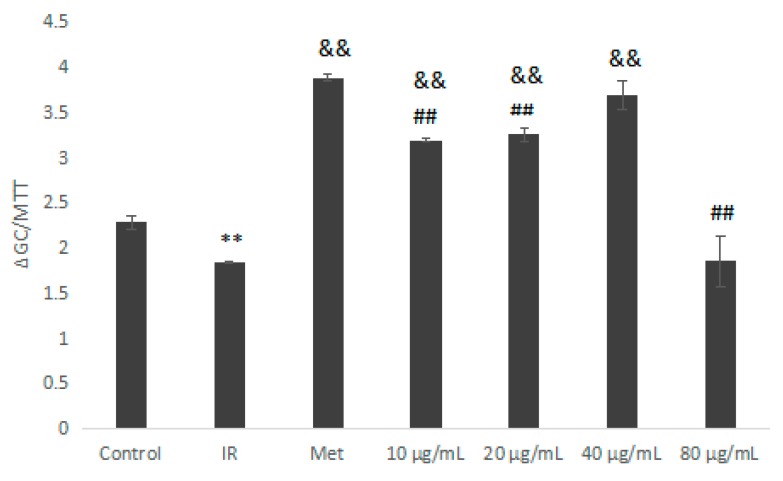
Effect of IOEP1 on the glucose consumption of insulin-resistance (IR) HepG2 cells. IR compared with the control, ** *p* < 0.01; compared with IR, ^&&^ *p* < 0.01; compared with the Met group, ^##^ *p* < 0.01.

As shown in Figure 9, we used 10^−6^ mol/L insulin and Met as the negative control and positive control, respectively. The glucose consumption of the insulin-resistance (IR) group was significantly lower than that of the normal control group (*p* < 0.01). Compared with the IR group, the Met group (10^−6^ mol/L) and the IOEP1 sample group (10–80 μg/mL) promoted glucose consumption in IR cells, and IOEP1 samples had the best effect on promoting glucose consumption at a concentration of 40 μg/mL, while the effect on promoting glucose consumption at 80 µg/mL was lower, which might be attributed to the high concentration.

**Figure 10:**
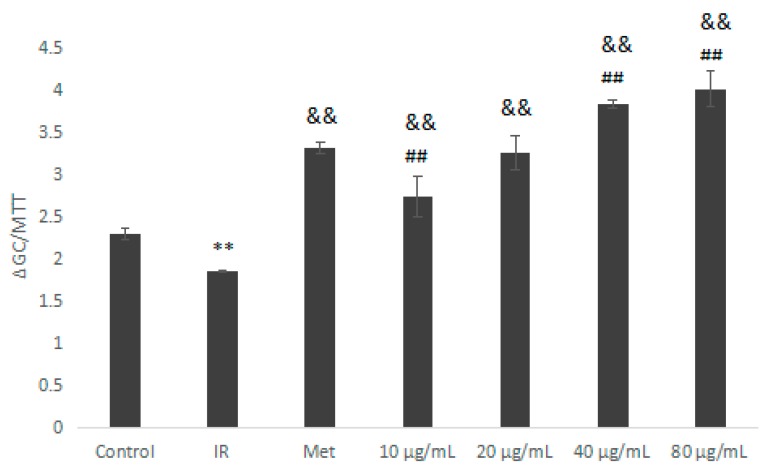
Effect of IOEP2 on the glucose consumption of IR HepG2 cells. IR compared with the control, ** *p* < 0.01; compared with IR, ^&&^ *p* < 0.01; compared with the Met group, ^##^ *p* < 0.01.

Compared with the IR group, the IOEP2 sample group (10–80 μg/mL) significantly promoted glucose consumption in IR HepG2 cells (Figure 10; *p* < 0.01). At an IOEP2 concentration of 80 μg/mL, the promotion effect was the best, which was significantly higher than that of the Met group (*p* < 0.01).

This indicates that certain concentrations of IOEP1 and IOEP2 could improve glucose consumption in IR HepG2 cells. The best glucose consumption in IR HepG2 cells occurred with IOEP1 at a concentration of 40 µg/mL and with IOEP2 at a concentration of 80 μg/mL.

## 3. Materials and Methods

### 3.1. Chemicals and Regents

*I. obliquus* strains were purchased from the Sichuan Institute of Edible Fungi. The DEAE-52 column was purchased from the Pharmacia Chemical Co. (Charlotte, NC, USA), and monosaccharide standards and insulin were purchased from Sigma-Aldrich Co. (St. Louris, MO, USA). Foetal bovine serum (FBS), Dulbecco’s modified Eagle medium (DMEM), penicillin, streptomycin, and phosphate-buffered saline (PBS) were purchased from Beijing Solarbio Science & Technology Co., Ltd. (Beijing, China). A glucose test kit was purchased from Shanghai Rongsheng Biotech Co., Ltd. (Shanghai, China). All other chemicals and solvents were of analytical grade.

### 3.2. Liquid Culture

*I. obliquus* was maintained on potato dextrose agar (PDA) slants at 4 °C for further use. *I. obliquus* was first grown on PDA medium in petri dishes for seven days. A 0.5 cm disk from the edge of the dish was cut out and inoculated in an Erlenmeyer flask containing 100 mL of medium. The composition of resting-cell transformation medium was optimized according to previous studies in our laboratory, and included 20 g/L glucose, 4 g/L tryptone, 1 g/L KH_2_PO_4_, 1g/L MgSO_4_, and 0.5 g/L MgCl_2_. The experiments were carried out with a shaking speed of 150 rpm at 28 °C for seven days. The fermentation medium was inoculated with 10% seed culture and then cultured in a 1 L Erlenmeyer flask. The compositions of growing-cell transformation medium consisted of 20 g/L glucose, 4 g/L tryptone, 1 g/L KH_2_PO_4_, 1 g/L MgSO_4_, 0.5 g/L MgCl_2_, and 4 mg/L VB6. The fermentation conditions were the same as those of the seed medium.

### 3.3. Extraction, Isolation and Purification of Polysaccharides

After the removal of mycelia by filtration, the culture broth on day 13 was concentrated up to a certain volume under vacuum, and then absolute ethanol was added to the final concentration (4:1, *v*/*v*) and stored at 4 °C overnight for 24 h. The precipitate was collected after centrifugation (4000 rpm/min, 10 min) and dissolved in distilled water, followed by the addition of Sevag reagent. The mixed solution was placed in a table concentrator and shaken for 30 min. The solution was left static for 30 min, after which supernatants were collected, lyophilized, and designated as IOEP. The polysaccharides after alcohol precipitation were freeze-dried to obtain crude polysaccharides [31]. 

The crude polysaccharide (IOEP) was dissolved in distilled water and passed through a 0.45 µm filter to remove impurities. The sample was applied to a well-balanced DEAE-cellulose column (2.6 × 50 cm), first eluted with distilled water, and then with 0.5 mol/L NaCl, respectively. The eluate was collected at a flow rate of 2.00 mL/min. The two collected IOEP fractions were denoted as IOEP1 (IOEP washed with distilled water) and IOEP2 (IOEP washed with 0.5 mol/L NaCl) for further research.

### 3.4. Analysis of Chemical Composition

For the determination of polysaccharides, the phenol sulfuric acid method was used. Protein content was determined by the Coomassie brilliant blue method.

### 3.5. Determination of Homogeneity and Molecular Weight

Purity and molecular weight were determined by GPC [32]. Standard dextran (T-180 Da, 5250 Da, 9750 Da, 13,050 Da, 36,800 Da, 64,650 Da, 135,350 Da) was used as a standard, and the sample to be tested was formulated into a solution using a high-performance GPC column (with Agilent PL aquageL-OH MIXED-M). A refractive index detector was injected with 20 μL, using 0.1 mol/L sodium nitrate buffer as the mobile phase and a flow rate of 1.0 mL/min. The retention time was determined at a column temperature of 30 °C. The average Mw (average molecular weight) values of the polysaccharides were determined on a standard curve.

### 3.6. Monosaccharide Composition Analysis

Ten milligrams of polysaccharide sample was hydrolyzed for 3 h at 105 °C with trifluoroacetic acid (TFA, 2 M) in a sealed tube. 1-Phenyl-3-methyl-5-pyrazolone (PMP) derivatization of the monosaccharides was carried out following a reported method with some modification [33]. Briefly, 1 mL hydrolysed polysaccharide sample or monosaccharide standard solution was mixed with 0.3 mol/L sodium hydroxide solution (600 µL). To the mixture, 0.5 mol/L methanol solution of PMP (600 µL) was added, followed by mixing using a vortex mixer. The mixture was kept at 70 °C for 2 h in a thermostat water bath. Finally, the mixture was neutralised with 0.3 mol/L hydrochloric acid (600 µL) after being cooled to room temperature. Then, 1 mL chloroform was added to the mixture and shaken vigorously. The extraction process was repeated five times to remove PMP. The aqueous layer was filtered through a 0.22 µm filter for HPLC analysis. The standard stock solution consists of seven monosaccharide standards of glucose, rhamnose, galactose, arabinose, xylose, mannose, and fucose. The HPLC system was coupled to a UV detector (250 nm) and an Agilent Eclipse-C18 column (250 × 4.6 mm), maintained at 30 °C. The column was eluted with an isocratic mobile phase consisting of acetonitrile and 20 mmol/L phosphate buffer solution (pH 6.7, 18:82, *v*/*v*) at 1 mL/min.

### 3.7. FT-IR Analysis

Infrared spectroscopy has the advantages of not destroying the sample, being high sensitive, and requiring low amounts of the sample for analysis. It is commonly used to analyze pyranose and furanose and to determine the configuration of glucosidic bonds in pyran polysaccharide. Other functional groups such as substituted groups are routinely observed. Quantitative analysis of polysaccharides is crucial for studying the structure of substituted polysaccharides, where it is difficult to find suitable solvents. The two polysaccharide were kneaded into KBr pellets for the measurement in a range of 4000 cm^−1^ to 400 cm^−1^ by the Nicolet Nexus FT-IR spectrometer [34,35].

### 3.8. ^1^H-NMR Analysis

The NMR method is widely used for determining position of glycosidic bonds, the order of monosaccharide residues, the number of monosaccharides in the repeated structure, and the types of α- and β-isomers without destroying the polysaccharide structure. Dried powders of the two types of *I. obliquus* polysaccharides were dissolved in D_2_O at a concentration of 10 g/L in a 5 mL NMR tube, and ^1^H-NMR spectroscopy was performed using an NMR spectrometer at 50 °C [36,37].

### 3.9. Ultraviolet Spectrum

IOEP1 and IOEP2 polysaccharide samples were weighed, dissolved in distilled water to a concentration of 0.1 mg/mL, and then measured at 25 ° C in a UV scanner with a wavelength of 200–400 nm to obtain an ultraviolet scan [32].

### 3.10. Hypoglycaemic Activity Assay In Vitro

#### 3.10.1. Cytotoxicity Assay on HepG2 Cells

The cytotoxic activity of IOEP1 and IOEP2 was determined in HepG2 (a human hepatoma cell line) cells by the 3-(4,5-dimethyl-2-thiazolyl)-2,5-diphenyltertazolium bromide (MTT) [38] assay. HepG2 cells were cultured in DMEM supplemented with 10% FBS and penicillin (100 U/mL)/streptomycin (100 µg/mL). The cells were maintained in an incubator at 37 °C and 5% CO_2._

The cells in the logarithmic growth phase were digested with 0.25% trypsin solution. The cell density was adjusted to 2 × 10^4^ /mL with the culture medium, and then cells were seeded in a 96-well cell culture plate at 200 μL/well at 37 °C and 5% CO_2_ condition. Next, cells were treated with IOEP1 and IOEP2 at 10, 20, 40, 80, and 160 μg/mL, respectively, for 24 and 48 h. Cell viability was determined by the ratio of absorbance at 570 nm. The effect of the two polysaccharides on cell viability was calculated using the following formula: percent viability = A_570nm_ of the treated sample/A_570nm_ of the untreated sample × 100%.

#### 3.10.2. Glucose Consumption Assay on HepG2 Cells

After the cells were cultured to the logarithmic period, they were routinely digested. Next, 200 μL of the cell suspension (2 × 10^4^ /mL) was added to a 96-well cell culture plate and cultured at 37 °C and 5% CO_2_. After the cells were attached, the old medium was aspirated, and serum-free DMEM medium was added to synchronise the cells. After 16 h of culture, the supernatant was aspirated, and serum-free drug-containing or drug-free DMEM was added. The experiment was divided into the following groups: blank control group, Met group (1 × 10^−3^ mmol/L), insulin group (10^−5^ mmol/L), and polysaccharide treatment group (10, 20, 40, 80 μg/mL). After 24 h of culture, the glucose content was determined at 505 nm according to the instructions of the glucose test kit, and the glucose consumption rate was calculated based on the following formula:

ΔGC/MTT = (glucose concentration of blank wells − glucose concentration of cell cell-inoculated)/MTT

#### 3.10.3. Glucose Consumption Experiment on Insulin-Resistance (IR) HepG2 Cells

The IR model was established by the high glucose and high insulin method [39]. The HepG2 cells in the logarithmic growth phase were digested with 0.25% trypsin solution, adjusted to a cell density of 2 × 10^4^ /mL, and seeded in a 96-well cell culture plate at 200 μL/well at 37 °C and 5% CO_2_. After the cells were attached, freshly prepared insulin-containing medium (final insulin concentration of 10^−6^ mol/L) was added, and a blank hole without insulin was placed as blank control and incubated at 37 °C in a 5% CO_2_ incubator for 24 h. Finally, the glucose content was measured using a glucose kit, and the establishment of the IR cell model was judged by the glucose consumption of the cells.

### 3.11. Statistical Analysis

All results are expressed as means ± SD. Statistical significance was tested by one-way analysis of variance (ANOVA) using SPSS 17.0, and *p*-values of less than 0.05 or 0.01 were considered statistically significant.

## 4. Conclusions

In the present study, the crude polysaccharide from *I. obliquus* in submerged culture was fractionated through DEAE-52 cellulose. Two major polysaccharide fractions (IOEP1 and IOEP2) were obtained. The chemical and physical properties of these polysaccharides were determined using chemical methods, including GPC, FT-IR, and HPLC. The hypoglycaemic activities were also evaluated in vitro. The results revealed the basic structural characteristics of two promising polysaccharides, IOEP1 and IOEP2. Furthermore, we highlighted a theoretical basis for these two polysaccharides as new hypoglycaemic drugs in the future. In the path ahead, the hypoglycaemic activity of IOEP1 and IOEP2 in vivo needs to be validated to ascertain their potential applications in natural hypoglycaemic drugs. Further studies to investigate the relationship between the structure and the hypoglycaemic activity are also warranted.

## Figures and Tables

**Table 1:** Major chemical composition of polysaccharides.

Sample	IOEP1 (%)	IOEP2 (%)
Carbohydrate (%)	87.04 ± 1.33 ^a^	78.73 ± 1.57 ^b^
Protein (%)	0.831 ± 0.73 ^a^	1.521 ± 0.34 ^b^

Means ± SD values are shown. Different letters in the same line indicate significant differences, *p* < 0.05.

**Table 2:** Toxicity of different concentrations of IOEP1 and IOEP2 on HepG2 cells.

	Concentration (μg/mL)	Cell survival rate (%)	
		IOEP1	IOEP2
Control Metformin Insulin	-	100	100
	-	100.1 ± 2.31	100.1 ± 2.31
	-	98.40 ± 4.69	98.40 ± 4.69
	10	97.74 ± 0.10	97.69 ± 2.05
	20	94.43 ± 1.54	95.88 ± 4.48
	40	93.77 ± 4.26	95.29 ± 1.66
	80	90.78 ± 2.01	93.45 ± 1.23
	160	87.90 ± 2.01	92.11 ± 1.68

The values are presented as means ± SD.
